# Referral of Touch and Ownership between the Hands and the Role of the Somatosensory Cortices

**DOI:** 10.1371/journal.pone.0052768

**Published:** 2013-01-02

**Authors:** Michael Schaefer, Franziska Konczak, Hans-Jochen Heinze, Michael Rotte

**Affiliations:** Department of Neurology, Otto-von-Guericke University Magdeburg, Magdeburg, Germany; University of Reading, United Kingdom

## Abstract

Recent studies have shown that the feeling of body ownership can be fooled by simple visuo-tactile manipulations. Perceptual illusions have been reported in which participants sense phantom touch seen on a rubber hand (rubber hand illusion). While previous studies used homologous limbs for those experiments, we here examined an illusion where people feel phantom touch on a *left* rubber hand when they see it brushed simultaneously with brushes applied to their *right* hand. Thus, we investigated a referral of touch from the right to the left hand (across the body midline). Since it is known from animal studies that tactile illusions may alter early sensory processing, we expected a modulation of the primary somatosensory cortex (SI) corresponding to this illusion. Neuromagnetic source imaging of the functional topographic organization in SI showed a shift in left SI, associated with the strength of the referral of touch. Hence, we argue that SI seems to be closely associated with this perceptual illusion. The results suggest that the transfer of tactile information across the body midline could be mediated by neurons with bilateral tactile receptive fields (most likely BA2).

## Introduction

In everyday life we do not question what belongs to our body and what not. Nevertheless, we know that simple tactile manipulations may induce profound tactile illusions. One of the oldest tactile illusions is the Aristotle illusion. It requires crossing the fingers and then touching the nose, which results in the feeling to have two noses [1]. This illusion arises because the brain fails to take into account that we have crossed the fingers. Since the nose touches the outside of both fingers at the same time (what rarely happens), our brain interprets it as two separate objects. In the last years numerous studies reported similar tactile illusions based on comparable visuo-tactile manipulations. For example, Lackner [2] reported an experiment in which the participants felt distorted body parts. The blindfolded participant was sitting at a table with his arm flexed at the elbow and holding the tip of his own nose. Now, the experimenter was using a vibrator to stimulate the tendon of the biceps. The subject was not only feeling that his arm has been extended, but also that his nose has been lengthened, making the participants feel like Pinocchio. Another prominent visuo-tactile illusion has been reported by Botvinick and Cohen [3]. They instructed participants to watch a (right) rubber hand placed on a table in front of them while their real right hand was hidden. Then, the experimenter touched both the real and the rubber hand with a small paintbrush. After a few seconds, the participants reported an astonishing feeling. The participants had the sensation as if the rubber hand was part of their own body. The illusion disappeared when a small asynchrony was introduced between the stroking of the rubber and the real hand [3], [4].

A recent study now reports an intriguing new version of the rubber hand illusion. Petkova and Ehrsson [5] found that healthy participants experience phantom touch on a *right* rubber hand when they see it brushed simultaneously with brushes applied to their *left* hand. Thus, they reported a referral of touch and ownership from the left to the right hand (across the body midline).

In our previous paper we similarly manipulated visual and tactile information in order to induce referred sensations in healthy participants [6]. While the original rubber hand illusion is an example for the referral of a somatic sensation off the body to an exterior artificial limb, we were interested in an illusion of a referral of a somatic sensation on the same body surface. Thus, we tried to induce a referred sensation in healthy individuals similar to that reported in phantom limb patients [7], [8]. Participants were stimulated on their fifth digit (D5) while watching a video that showed a life-sized hand stimulated at the first digit (D1). Hence, we induced a conflict in feeling and seeing. Participants reported a referred sensation of feeling the stimulation on D1 instead of D5 when the stimulation was in-phase with the video. Brain imaging results revealed a modulation in SI corresponding to this illusion. We concluded that SI seems to be involved in this referral of touch. However, whereas our study examined a referral of touch from one finger to another one on the same hand, Petkova and Ehrsson [5] reported a referral of touch from the left to the right hand. Thus, the referral of touch in their study included not only a much bigger distance, it also crossed the body midline.

What may be the neural mechanisms of these illusions of body ownership and referral of touch? According to an fMRI study by Ehrsson and colleagues the premotor cortex may reflect the feeling of ownership of a seen hand, whereas parietal areas might play a role in integration of arm orientation and binding of visuo-tactile events [4]. Furthermore, somatosensory areas have been related to body ownership illusions (e.g., [6]. However, it is not clear which brain areas were engaged during the referral of touch Petkova and Ehrsson [5] described. Since previous work highlighted the role of somatosensory areas for experimentally induced referred sensations [6] or for referred phantom feelings [7], [8], we hypothesized that SI may play an important role in this referral of touch.

Hence, based on animal (e.g., [9]) and fMRI studies (e.g., [10]), which demonstrated close relationships of tactile illusions with SI, the results of our previous study [6], and reports on referred sensations subsequent upper limb amputation [7], [8], we assumed that the referral of touch reported by Petkova and Ehrsson [5] is linked to analogue modulations in SI. However, the study by Petkova and Ehrsson [5] demonstrated a referral of touch across the body midline. This raises the question about the contribution of bilateral somatosensory cortices to this illusion.

The present study aimed to test the hypothesis of an involvement of the somatosensory cortices to this illusion and to disentangle the contribution of ipsi- and contralateral somatosensory cortices. To this end we replicated the experiment of Petkova and Ehrsson [5] while recording neuromagnetic fields in order to assess possible changes in SI. The results would give important insights about the neural mechanisms of this bimanual referral of touch.

## Materials and Methods

### Participants

Twenty-seven right-handed participants (15 females) with a mean age of 25 years (range 22–27 years) participated in the study. All of the 27 participants took part in the behavioral parts of the study (questionnaire and pointing task). In the neuromagnetic part of the experiment five participants (right hand; left hand: seven participants) had to be excluded due to poor goodness of fit. Finally, using only participants that feel the illusion, 11 participants (for the right side, 10 for the left side) of this sample were included for subsequent statistical analysis.

The participants gave informed written consent to the study, which adhered to the Declaration of Helsinki and was approved by the human participants committee of the Otto-von-Guericke University Magdeburg.

### Procedure

Participants were seated on a comfortable chair inside a magnetically shielded room with their head placed in the mould of the dewar of the whole-head magnetoencephalography (MEG) system (4D Neuroimaging, San Diego, CA, USA). To support fixation of the participants' head, we used small cushions placed in the gap between the head and the mould of the dewar.

**Figure 1:**
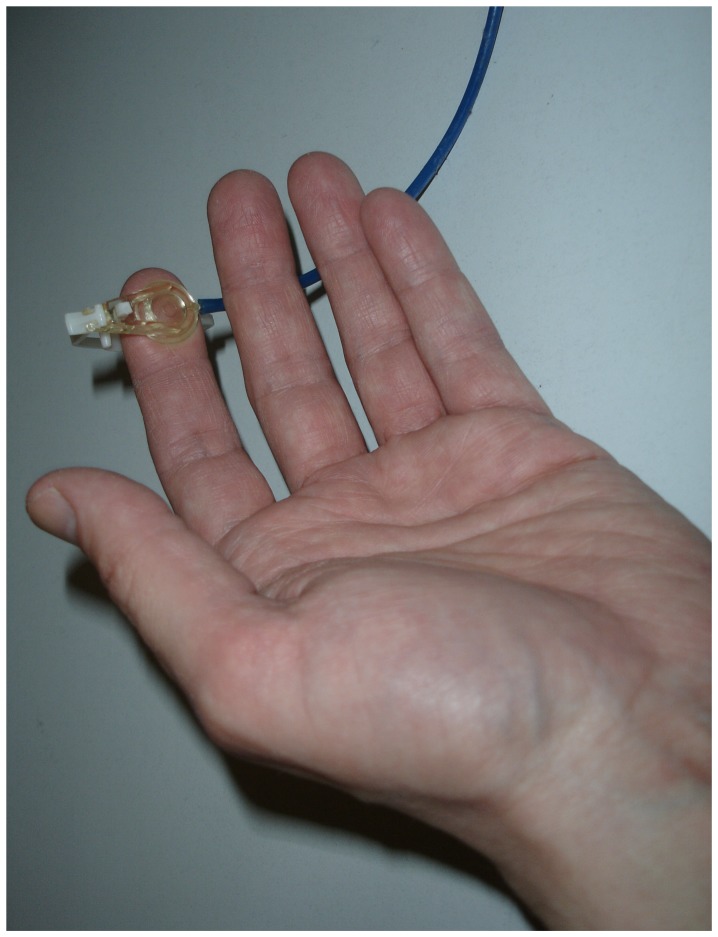
Pneumatical stimulation device.

In order to map the functional topography in SI, we applied tactile stimuli at the distal phalanges of D2 and D5 of the left and right hand using a pneumatically driven stimulator (4D Neuroimaging, San Diego, CA, USA) (Fig. 1). The stimulation device consisted of a diaphragm with a 10 mm diameter causing a distinct tactile sensation when inflated toward the skin by a pulse of pressed air of 2.5 atm for 20 ms.

During the experimental blocks we adopted the altered rubber hand paradigm by Petkova and Ehrsson [5]. The participants put their arms in a resting position on a table. A life-size left rubber hand was placed on the table 21 cm to the left of the midline of the participants' body. The participants' real left hand was hidden behind a screen at a distance of 20 cm from the rubber hand. The participant's right hand was placed in full view 21 cm to the right of the midline of the body, giving the participant the visual impression that he had placed both of his hands on the table parallel to one another (the proximal ends of the arms were covered by a blanket). The participants were instructed to focus the rubber hand. An experimenter now stroked the real right hand and the left rubber hand with two (identical) paintbrushes either in in-phase (synchronously) or out-of phase (asynchronously) (for about one second). We expected that in the in-phase condition the rubber hand illusion would be elicited, while the out-of-phase condition provided the control condition.

In each session the participants' right hand was touched with a paintbrush either synchronously or asynchronously to the paintbrush stimulation of the (left) rubber hand (independent of the pneumatical stimulation). The experiment consisted of four experimental blocks: Pneumatic tactile stimulation of the fingers of the left hand during synchronous paintbrush stimulation with the rubber hand (left synchronous condition), pneumatic tactile stimulation of the left hand during asynchronous paintbrush stimulation with the rubber hand (left asynchronous condition), pneumatic tactile stimulation of right hand during synchronous paintbrush stimulation with the rubber hand (right synchronous condition), and pneumatic tactile stimulation of the right hand during asynchronous paintbrush stimulation with the rubber hand (right asynchronous condition). The touches of the paintbrushes were delivered to the index fingers. In addition to those four experimental blocks two resting blocks were included (rest condition). In these resting blocks the participants received pneumatical stimulation of D2 and D5 (one block for left hand, another block for right hand), were told to relax and to gaze at the rubber hand. Here, neither the rubber hand nor the real hand received any paintbrush stimulation. However, the experimenter was present even in this resting block. All blocks were presented in a randomized order.

**Figure 2:**
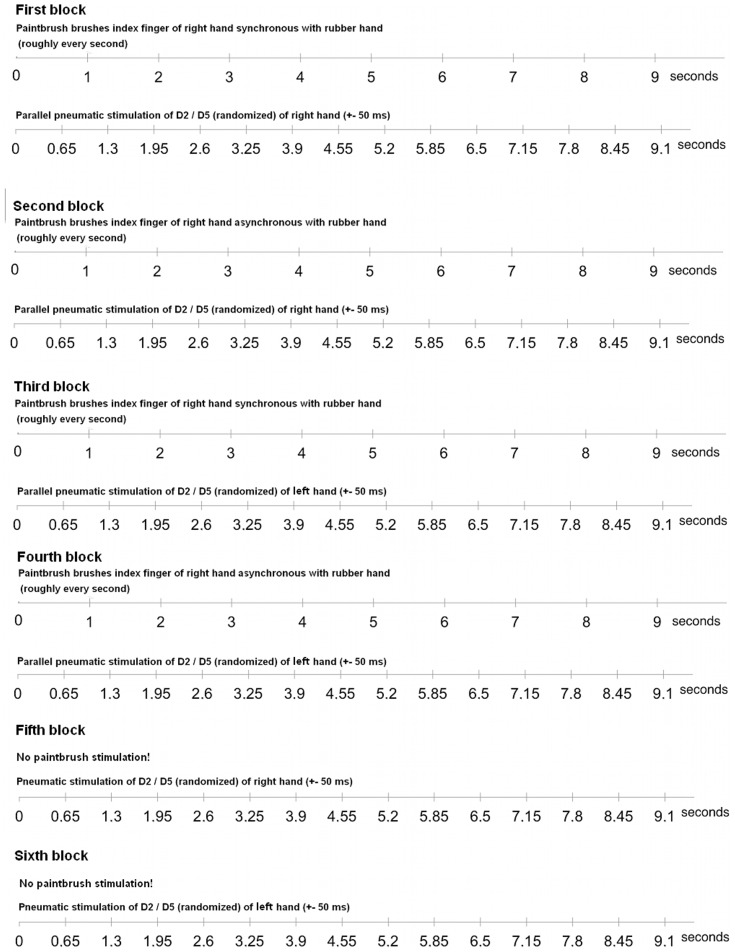
Timing of pneumatic and brushing stimulation. Note that the brushing of rubber and real hand was only roughly about one second. Similar, the pneumatic stimulation varied +−50 ms. Moreover, the experimenter was not able to notice the beginning of the pneumatic stimulation. Thus, there was always a randomized delay between the timeline of pneumatic stimulation and the timeline of the brushing by the experimenter (in contrast to the figure).

In each block (experimental and resting blocks) D2 and D5 were pneumatically stimulated; with each block lasting for approximately 10 minutes. Each finger received 400 stimuli resulting in 800 stimuli for each experimental block. Stimuli were presented with an interstimulus interval of 650±50 ms (Fig. 2). Participants were instructed to ignore all pneumatical tactile stimuli and not to move their head. All participants were highly used to tasks including pneumatical stimulation. None of the participants stated to have difficulties ignoring the tactile stimulation device. Eye and head movements of the participants were monitored online with a video camera. In addition, head movements were recorded by a 3D-digitizer at the beginning and at the end of each block. Participants demonstrating head movements were asked to replicate the experimental block or were discarded from the experiment.

Both before and immediately after the blocks, the participants completed a series of three intermanual reaches in order to measure the drift in the perceived location of the hand towards the location of the rubber hand. This drift provides objective behavioral evidence that the rubber hand is perceived as one's one hand [3], [5]. The participants were asked to close their eyes and indicate the position of their left index finger by pointing with their right hand. The experimenter asked the participant to move his right index finger briskly along a ruler and stop until it was above where he felt the left hand to be located. This task was repeated three times. We calculated the differences in pointing error towards the rubber hand (mean displacement) before and after each brushing session.

Subsequent to the pointing task we asked the participants to complete a questionnaire in which they had to indicate the occurrence of specific perceptual effects they had experienced. All questions were asked both for the synchronous and the asynchronous conditions. The questionnaire included nine statements. The first four questions were related to the sensation of touches on the rubber hand and the feeling of ownership of the hand. The fifth statement was to explore possible sensations in the real left hand. The remaining four statements served as control questions. The statements are depicted in Table 1. Participants indicated their response on a seven-point scale ranging from ‘disagree completely’ to ‘agree strongly’ (Likert scale). In order to avoid movements of the participant, the questions were read by the experimenter. Participants responded by telling the degree of confirmation to the statements.

**Table 1:** Questionnaire results (after synchronous brushing).

Statements	Yes	Uncertain	No
1. I felt as if the rubber hand was my hand.	10	11	6
2. It seemed as though the touch I felt was caused by the paintbrush touching the rubber hand	8	-	19
3. It seemed as if I was feeling the touch of the paintbrush on the rubber hand	5	5	17
4. I could sense two touches, both on my (real) right hand and on the left rubber hand	6	3	18
5. I had (weak) sensations of tingling/prickling/tickling or touch in my real left hand	10	1	16
6. It seemed as if I had two right hands or arms	2	1	24
7. It seemed as if the touch I was feeling came from somewhere between my own right hand and the rubber hand	2	1	24
8. It felt as if my (real left) hand were turning ‘rubbery’	3	2	22
9. The rubber hand began to resemble my own (real left) hand, in terms of shape, skin tone, freckles or some other visual feature	17	1	9

### Magnetic source imaging

Recording of somatosensory evoked magnetic fields (SEFs) were carried out with a whole head MEG-system with 148 first-order gradiometers. The MEG data were acquired with a sampling rate of 2034 Hz and high-pass filtered at 0.1 Hz. Using a trigger signal that was recorded simultaneously at the onset of the pneumatic stimulation, the MEG record of each trial was epoched into 400 ms windows. Somatotopic representations of the stimulated fingers were determined by source modeling of the earliest prominent activity peak of the magnetic brain response ranging in a time window from 35 to 85 s (M60 component) [6], [11], [12], [13]. The generator of the M60 component has been related to neural sources in SI by previous work (e.g., [14]). Furthermore, we performed source modeling for the secondary somatosensory cortex (SII), based on the second prominent peak (time window 85 to 150 s) [14]. The dipole model explained at least 90% of the variance. Individual MR images (GE 1.5T scanner, 3D-SPGR, T1-weighted, TR = 24 ms, TE = 8 ms) were then used for overlay of the dipole localizations with the anatomic structure of the subject's cortex. To achieve the overlays and to determine the source localizations of the SEFs, CURRY multi-modal neuroimaging software (Neuroscan, El Paso, TX, USA) was employed.

Modulations of the topographical localization of SI were quantified using a distance measure between the equivalent dipole locations modeling the cortical representations of D2 and D5. We examined distances between dipole positions of D2 and D5 instead of analyzing absolute dipole positions because this measurement is more robust with respect to systematic localization errors [12]. Using polar coordinates, representational shifts along the postcentral gyrus were quantified by differences in polar angle Δθ between dipole locations corresponding to the stimulation of D2 and D5. Changes in the cortical finger representation in anterior-posterior and radial direction were expressed by either azimuth differences (Δφ) or by differences in eccentricity (Δr) of the different dipole positions (e.g., [12]). Changes in the amount of cortical activity due to different stimulation conditions were assessed by comparing the dipole moments corresponding to the stimulation of D2 and D5.

A repeated measurements analysis of variance (ANOVA) with the factor ‘condition’ (rest, asynchronous ( = control), synchronous ( = experimental)) was performed for statistical comparisons of differences of the cortical distances. Significance levels were adjusted with the Greenhouse-Geisser epsilon coefficient [15]. Dipole parameters were then subjected to t-tests for paired samples (two-tailed). Dipole parameters for sources in SII were analyzed in an analogue way. The behavioral data were analyzed with t-tests for paired samples (one-tailed). P-values less than 0.05 were considered statistically significant.

## Results

### Behavioral results: Questionnaire data

When the rubber hand was brushed in synchrony with the participant's right hand, 21 out of our 27 participants felt (or were at least unsure) as though the rubber hand was their real hand (rating≥0 for question 1) (mean ± standard deviation: −0.71±2.41). Eight participants felt as though the touch they felt was caused by the paintbrush touching the rubber hand (−1.10±2.02) (question 3: −1.23±1.90; question 4: −1.02±2.00; see Table 1 for details).

Statistical analyses revealed that rating scores on the illusion (question 1–4) were significantly greater in the synchronous condition compared with the control condition (asynchronous brushing) (question 1: t(26) = 2.11, p = 0.02; question 2: t(26) = 3.13, p = 0.002; question 3: t(26) = 2.24, p = 0.01; question 4: t(26) = 3.35, p = 0.001). The responses to the control questions were not different for synchronous or asynchronous stimulation (questions 5 to 9: p>0.10). Furthermore, the rating scores on the illusion (question 1 to 4) were significant greater compared with the control questions (mean of questions 1 to 4 compared with questions 6 to 8; question 6: t (26) = 4.22, p = 0.0005; question 7: t (26) = 3.58, p = 0.0005; question 8: t (26) = 2.36, p = 0.01). Similarly to the results of Petkova and Ehrsson [5], question 9 was answered positively by most of the participants (17 participants confirmed this statement, see Table 1).

The rating scores were not different for right and left hand pneumatical stimulation (comparison of means of question 1 to 4, p>0.10).

### Behavioral results: Proprioceptive drift measure

Our results demonstrated a drift in the perceived location of the left hand towards the rubber hand in the illusion condition (synchronous brushing). The pointing error was significantly greater after the synchronous than after the asynchronous condition (synchronous: 0.78 cm; asynchronous: 0.33 cm; t (26), t = 1.90, p = 0.03). The rating scores were not different for right and left hand pneumatical stimulation (comparison of means of question 1 to 4, p>0.10).

### Neuromagnetic source localization

Since the behavioral data demonstrated that only half of the participants felt the illusion (similar to [5]), the neuromagnetic data analysis included only participants that felt the illusion.

**Figure 3:**
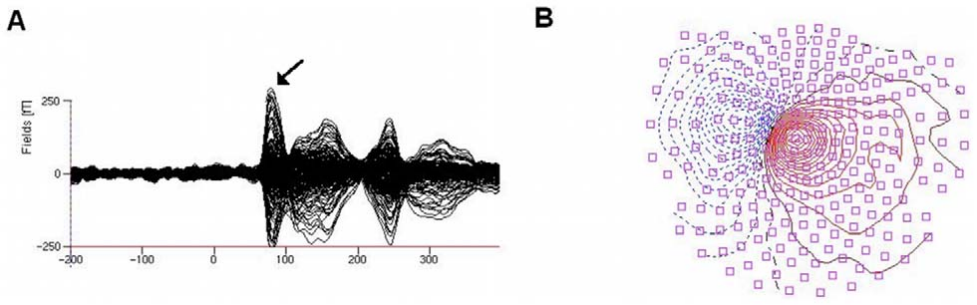
Waveform of magnetic activity and topographic map evoked by stimulation of the right D5 (representative subject, rest condition). Time courses of single MEG channels are superimposed from 148 sensors. Isocontour maps show the magnetic potential pattern at the first prominent peak (SI; see arrow) after stimulus onset (nasion up, right side displays the right hemisphere, left side the left hemisphere).

The neuromagnetic data for the first prominent peak revealed a clear dipolar neuromagnetic response in the contralateral hemisphere of each participant. The source localization of these dipoles could be well modeled in the region of the central sulcus contralateral to the stimulated side, as verified by overlay onto the magnetic resonance image. An example of the time course of the evoked magnetic activity and the corresponding scalp topography is shown in Figure 3.

**Figure 4:**
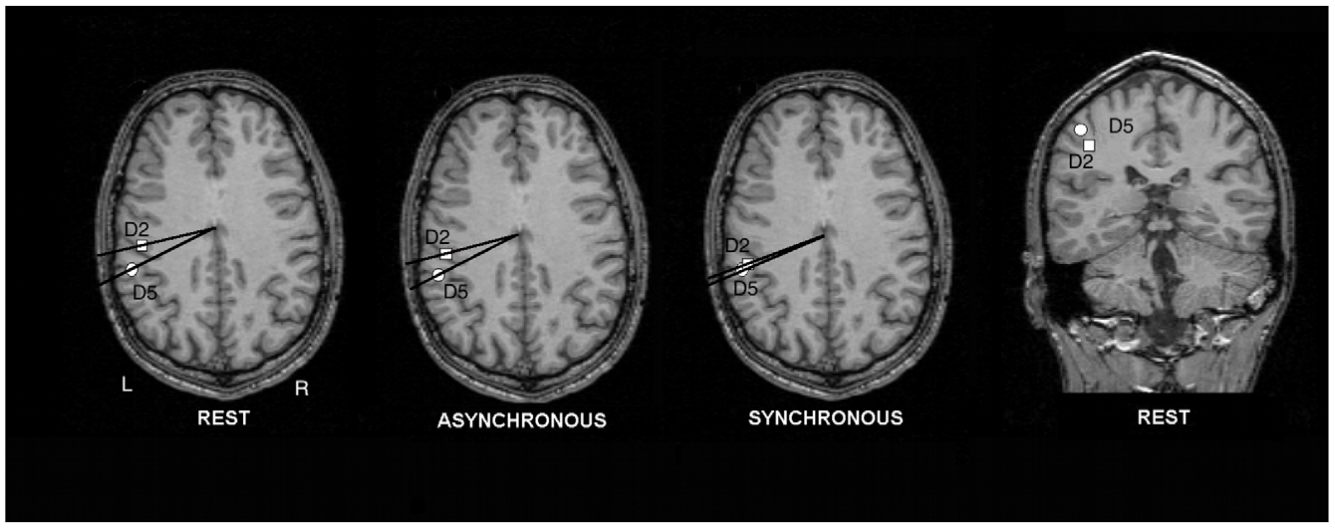
Dipole localizations of the SEFs for one representative subject overlaid onto an axial MRI slice. The positions of the dipole sources are specified in polar coordinates. The squares depict the cortical representations of D2; the circles show the representations for D5. Note the differences in polar angle Δφ between rest, asynchronous, and synchronous brushing, pointing to a decrease of the cortical distance in the synchronous brushing condition. For verification of dipole sources a coronal slice (rest condition) is depicted.

**Figure 5:**
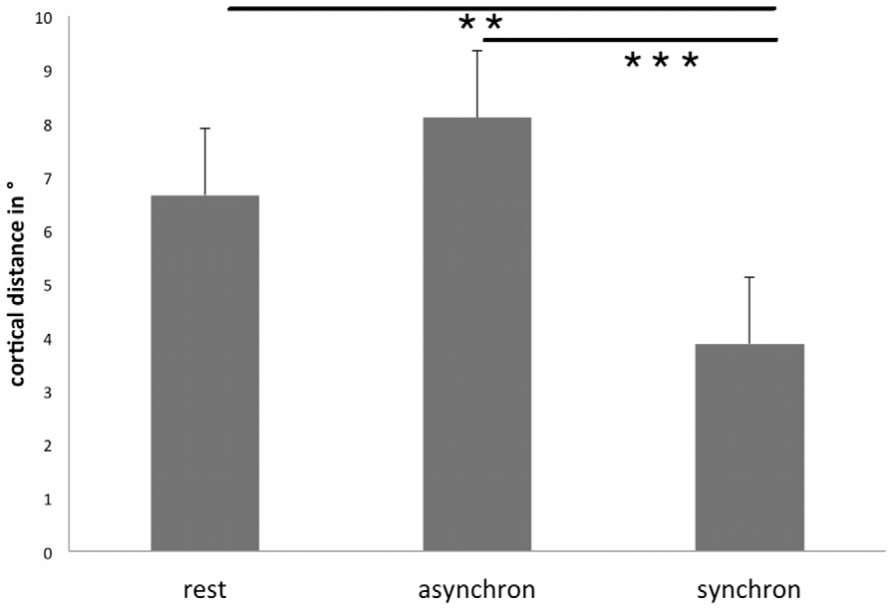
Group means and standard error for the cortical distance between the representations of D2 and D5 (in polar angle Δφ, in °) for the right hand. Asterisks indicate a significance decrease of the polar angle Δφ of the cortical representation for the right hand in the synchronous condition relative to rest and control (** = p≤0.01; *** = p<0.005).

A statistical test (ANOVA with factor condition: rest, synchronous brushing, asynchronous brushing) for changes of the cortical representations on the right hand revealed a main effect for condition (polar angle Δφ; F(2;20) = 7.53; p = 0.004). Post-hoc t-tests showed that the polar angle Δφ between the representations of D2 and D5, serving as a distance measure of the cortical hand representation in the anterior-posterior direction, was significantly smaller in the illusion condition (3.88±2.38°, mean ±SD) compared with rest (6.66±4.90°; t(10) = 2.45, p = 0.01) and compared with control (asynchronous brushing, 8.11±5.63°; t(10) = 3.43, p = 0.003). The comparison between rest and asynchronous brushing failed to show significant differences, as expected (t(10) = −1.55, p = 0.08). This demonstrates a modulation of left SI along the anterior-posterior dimension (see Figs. 4 and 5 and Table 2). We hypothesized that the representation of D2 shifted to a more posterior position (analogue to our previous paper [6]), resulting in a decreased cortical distance between both dipoles. In order to test this hypothesis, we compared the distance of the cortical representation of D2 during synchronous brushing and during resting position with the distance between D2 during asynchronous brushing and during resting position. Results yielded a significant difference (t (10) = 3.08, p = 0.005). In contrast, the analogue comparison for the cortical representation of D5 failed to show significant changes (p>0.10). Hence, the reduced distance between D2 and D5 during synchronous brushing seems to rely in particular on a modulation of the cortical representation of D2 towards a more posterior position. This posterior shift may indicate that BA 2 has become activated in addition to BA3b (similar to our previous paper [6]).

**Table 2:** Results of the neuromagentic source imaging and behavioral results (coordinates in x- -(medial-lateral), y- (anterior-posterior), z- (inferior-superior) dimensions) (for right hand; part. = participants; dist. = cortical distance, incl. = included).

part.	D2sync right hand	D5sync right hand	dist. in φ	Q1	Q2	Q3	Q4	drifts in cm	incl. in MEG illusion group
1	-	-	-	2	−1	0	2	1.00	No
2	41.9, −16.3, 123.3	42.8, −20, 131.2	3.79	−3	−3	−3	−3	1.34	No
3	52.2, −22, 115.6	51, −16.9, 115	4.52	−2	0	0	−1	−0.34	Yes
4	39.1, −38.7, 115.8	34.9, −61.3, 113.6	15.6	−3	−3	−3	−3	−0.33	No
5	39.3, −38.1, 124.9	38.8, −42, 135.7	3.16	3	0	0	0	1.00	Yes
6	39.9, −28.7, 128.6	33.9, −27.5, 131.6	3.32	1	−3	0	−1	−1.33	Yes
7	47, −31.6, 100.4	47,2, −32.9, 101.4	0.96	−2	0	0	−1	0.67	No
8	34.8, −22.4, 123,2	38.2, −22,1, 125.7	2.72	−3	−3	−3	−3	0.33	No
9	-	-	-	−3	−3	−6	−3	1.00	No
10	-	-	-	2	1	1	1	0.33	No
11	34.8, −42.9, 98.6	37.3, −45.5, 99.4	0.36	3	2	−1	1	0.67	Yes
12	35.5, −22, 131.5	32.1, −14.6, 139.6	7.33	0	1	1	−3	1.00	No
13	44.8, −32.2, 113	35.9, −32.1, 108.8	6.10	−2	−3	−1	−2	−0.34	No
14	43.8,−22.8, 98.7	39.6, −20.2, 114	0.47	3	3	3	3	−0.33	Yes
15	-	-	-	3	0	1	1	10.33	No
16	40.8, −36.6, 124.7	49.2, −40.4, 126.3	2.50	3	3	3	3	0.33	Yes
17	42.6, −27.1, 120.8	48.9, −22.7, 117.3	7.56	1	−2	−3	−3	−0.33	Yes
18	43.3, −30.7, 111.5	50, −40.2, 126.9	3.46	−3	−3	−3	−3	1.67	Yes
19	39.5, −0.3, 125.1	41.1, 3.1, 127.8	4.75	−3	−1	0	−1	0.33	No
20	38.4, −23.3, 128.2	42.4, −17.1, 128.9	9.29	−3	−3	−3	−3	0.33	No
21	44.8, −33.9, 128.1	49.9, −32.6, 131.7	3.96	−3	−3	−2	−3	2.67	No
22	33.9, −1.6, 123.4	37.3, −5.3, 137.3	5.39	1	−3	−3	−3	0.00	Yes
23	-	-	-	−3	−3	−3	−2	−1.00	No
24	44.6, 2.4, 123.8	36.2, −0.8, 120.8	4.35	3	2	2	2	0.00	Yes
25	31.1, −45.5, 114.2	35.4, −44.5, 109.1	4.15	−3	−3	−3	−3	0.33	No
26	51.4, −37.2, 116.8	52, −24.6, 117.6	10.6	−3	−3	−3	−2	1.00	No
27	57.3, −27.9, 104.6	52.5, −17.5, 104.4	7.53	−3	−3	−3	−3	2.00	Yes
	mean (SD) for all participants included in MEG experiment	3.88 (±2.38	0.91 (±2.47)	−0.36 (±2.54)	−0.45 (2.38)	−0.45 (±2.42)	0.30 (±0.97)		
	mean (SD) for all participants not included in MEG experiment	6.30 (±4.19)	−1.69 (±2.15)	−1.88 (±1.59)	−1.69 (±2.02)	−1.75 (±1.69)	1.19 (±2.57)		
	mean (SD) for for all participants feeling the illusion (right hand)	3.39 (±2.42)	2.27 (±0.90)	0.18 (±2.23)	0.27 (±2.05	0.55 (±2.11)	1.06 (±3.15)		
	mean (SD) for for all participants not feeling the illusion (right hand)	6.05 (±3.79	−2.63 (±0.81)	−2.25 (±1.39)	−2.19 (±1.76)	−2.44 (±0.81)	0.67 (±0.96)		

Q1 to Q4 refer to the first four questions of the questionnaire, which point to the illusion (Likert-scale, from −3 to +3). Drifts refer to the pointing task (see text for further details).

**Figure 6:**
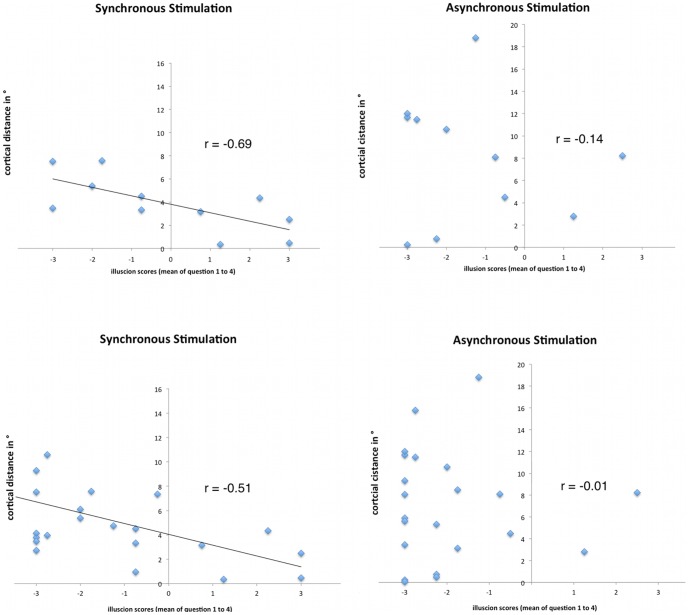
Scatter plots of the correlation between the modulations in left SI with the strength of the illusion (mean of questions 1 to 4, which indicate the illusion). The y-axis depicts the cortical distance between D5 and D2. Left part of the picture shows the scatter plots for synchronous stimulation, right side for asynchronous stimulation (control condition). The upper part depicts scatterplots for those subjects who felt the illusion, below the scatter plots for all subjects are shown. The data show significant correlations of the strength of the illusion with reduced cortical distances in SI (meaning a shift to posterior for D2) during synchronous stimulation. Thus, the stronger the participants felt the illusion, the more the source of D2 shifted towards posterior. In contrast, when stimulating the rubber hand asynchronously (right part of the picture), data analysis between illusion scores and modulation in SI revealed no significant correlations.

Furthermore, data analysis revealed that the shift in SI was significantly correlated with the feeling of the illusion (mean of questions 1–4: r = −0.69, p = .01; Pearson). For the control condition (asynchronous stimulation) there was no significant correlation with modulations in SI (r = −0.14, p = 0.68; Pearson). When not restricting the analysis to participants who felt an illusion (N = 22) the correlation was lower, but still significant for synchronous brushing (r = −0.51, p = 0.02; Pearson), whereas the control condition failed to show a significant correlation (asynchronous brushing, r = −0.01, p = 0.95, Pearson, see Fig. 6). Furthermore, the control questions (mean of questions 6–9, for synchronous stimulation) revealed no significant correlations with modulations in SI (p>0.10).

In contrast to the right hand, an ANOVA testing for representational changes for the left hand failed to show a significant effect for condition (polar angle Δφ; F(2;18) = 3.07; p = 0.10). Thus, the polar angle Δφ between the cortical representations of D2 and D5 was not significantly different across the conditions (synchronous brushing: 7.78±8.17°, asynchronous brushing: 6.83±8.21°; rest: 5.81±7.23°; rest vs. asynchronous: t(9) = −1.71, p = 0.12; rest vs. synchronous: t(9) = −1.86, p = 0.10; synchronous vs. asynchronous: t(9) = −1.47, p = 0.17).

No significant effects (ANOVA) were found in radial eccentricity (r) or along the postcentral gyrus (polar angle Δθ) of the dipole sources. Statistical analysis (ANOVA) of the dipole moments yielded no significant effects. No effects were found for right relative to left hand pneumatical stimulation. Furthermore, neuromagnetic analysis for SII revealed no significant effects.

## Discussion

The present study examined a new version of the well-known rubber hand illusion. Participants sense phantom touch on a left rubber hand while their *contralateral* (right) hand is brushed synchronously to the rubber hand at the corresponding homologue's site. Thus, the illusion describes a referral of touch and ownership from the right to the left hand. Our behavioral results replicated the findings of Petkova and Ehrsson [5]. Questionnaire data as well as the results of the proprioceptive drift measure provided subjective and objective behavioral evidence that the rubber hand was perceived as one's own hand. Neuromagnetic source imaging allowed examination of the contributions of bilateral somatosensory cortices to this illusion. The cortical representation of D2 of the left hemisphere demonstrated a change towards a more posterior position during the illusion, pointing to an additional involvement of BA2. This modulation in SI was associated with the strength of the illusion. Thus, the stronger the participants felt the rubber hand as their own hand, the greater was the magnitude of the shift in SI. In contrast, analysis of the somatosensory cortex in the right hemisphere yielded no modulation of dipole sources (or dipole strengths).

Questionnaire data as well as proprioceptive drift measures replicated the illusion first reported by Petkova and Ehrsson [5]. However, similar to the findings of Petkova and Ehrsson [5] less than half of our participants felt the illusion, whereas the original rubber hand illusion works in about 70% of the participants [16]. This may be explained by the requirements of additional processes related to the integration of visual and tactile input from the opposite sides of the body, as Petkova and Ehrsson [5] suggested. The differences in mean pointing error was significant for synchronous relative to asynchronous brushing, thereby providing objective results for the illusion. The absolute values in our study are somewhat lower than in the Petkova and Ehrsson [5] study, which might be explained by the higher number of participants that did not feel any illusion in our study. However, in contrast to the questionnaire data the proprioceptive drift showed significant results for all subjects. This may be explained by recent suggestions that the feeling of ownership and the proprioceptive drift may not go hand in hand but rely on different mechanisms of multisensory integration [17].

Previous results coming from animal experiments [9] and studies with fMRI or MEG [6], [10] demonstrated a close relationship of tactile illusions with SI. For example, our previous study on referred sensations from one digit to another (on the same hand] showed a significant correlation of the illusion with modulations in SI [6]. In addition, upper limb amputees with phantom limb feelings report referred sensations (even across the body midline, [18]), which have been discussed to be caused by modulations in SI [7], [8]. Based on those studies we hypothesized that the transfer of tactile information from the right to the left hand in the altered rubber hand illusion [5] is based on SI.

Data from neuromagnetic source imaging supported our hypothesis. The illusion was linked to alterations in left SI. This modulation in left SI correlated significantly with the strength of the illusion. In contrast, for right SI we found no effects. Thus, we conclude that left SI seems to be closely linked to this illusion. The results are in line with our previous study on referred sensations and SI [6], in which we reported a shift in SI towards a more posterior position (change of polar angle Δφ) that corresponded with the strength of the illusion. The current study demonstrates a similar shift in SI towards posterior, associated with the strength of the illusion. Analogue to our previous study [6], this posterior shift seems to point to an involvement of additional posterior areas for the illusion, most likely BA2.

The results support the hypotheses of Petkova and Ehrsson [5]. The authors hypothesized that extended stimulation of the subject's right hand may have elicited weak ipsilateral responses, which then would be combined with temporally and spatially congruent visual information from the contralateral rubber hand (probably via BA2 or BA5), resulting in an up-regulation of those ipsilateral responses and a feeling of the rubber hand. The authors linked their hypothesis with results from monkey single-neuron recordings indicating bilateral hand representation in the posterior bank of the postcentral gyrus (BA2) and along the border between BA2 and BA5/7 [19]. Petkova and Ehrsson [5] suggested that the transfer of tactile information from the right to the left hand in the illusion could be mediated by those neurons with bilateral tactile receptive fields. Neuromagnetic source imaging in our study revealed that left SI including most likely BA2 was involved when feeling the illusion. Tactile information of left SI may have been transferred to the right somatosensory cortices via BA2, resulting in the feeling of the rubber hand. BA2 is known to have the densest transcallosal connections among all SI areas [20]. Furthermore, BA2 has reciprocal connections to BA3b [21].

Since we did not find alterations in right SI, our results suggest a role for SI in particular for the hemisphere contralateral to the brushed (real) hand for this transfer of illusion. However, alternative explanations should also be taken into account. Thus, there might be a specific role for left SI in this transfer of illusion irrespective of the location of the rubber hand, as suggested by recent studies on vicarious somatosensory activation when observing touch [22]. The present study did not vary the location of the rubber hand in the visual field. Thus, we cannot exclude this explanation.

Numerous studies demonstrated that the functional topography of SI can be modulated without preceding long-lasting sensory training or deafferentation (e.g., [12]). This short-term plasticity is able to change somatotopic representations dynamically and task-dependently. Braun et al. [12] suggested specific representational maps that were activated by switching between stable maps established earlier, possibly related to sub-threshold synaptic activities [23]. What might be the neural mechanisms of the modulation in SI we report in the current study? In everyday life we often have to integrate information from different modalities, e.g., vision and touch. Previous research has demonstrated that this polymodal integration also works at the level of SI, most likely involving BA2 (e.g., [24]). BA2 receives input from BA3a,b and BA1. This information is combined with proprioceptive input from the thalamus. Moreover, BA2 has callosal connections with BA2 of the other hemisphere. These connections enable some neurons in BA2 to respond to stimuli of both ipsi- and contralateral hands. Furthermore, BA2 has also direct reciprocal connections with the ventral intraparietal area and the inferior parietal lobe, in which visual, somatosensory, and auditory information are combined. Interestingly, these areas project to the premotor cortex. Taken together, BA2 receives direct input from regions that are responsive to visual stimuli, whereas BA3b has only indirect access to such information [24]. Hence, we argue that the modulation in SI we report in the current study may indicate that BA2 has become activated in addition to BA3b.

A possible objection to our results may be the use of a pneumatic stimulation device for mapping changes in SI. However, none of our participants stated to have been irritated by the device. The pneumatic stimulation, which was applied below the finger, delivered pulses much faster and not related to the brushing with the paintbrush. Moreover, the short and brisk touches delivered by the pneumatic stimulation device felt different than the touches of the paintbrush. Since our participants participated in comparable studies before, they were highly used to this pneumatic stimulation and had no difficulties to ignore it. Furthermore, questionnaire and pointing data did not differ for left and right hand pneumatic stimulation. In addition, changes in SI were also independent from the pneumatic stimulation. Thus, we think that it is unlikely that the pneumostimulation device may have influenced the results.

The present experiment used neuromagnetic source imaging to assess changes in bilateral SI. Neuromagnetic source analysis can calculate dipole sources in the somatosensory domain with a very high reliability and validity [25], [26]. Previous studies have demonstrated that the precision with which neural generators can be calculated depends in particular on the signal-to-noise ratio of the evoked activity. It has been demonstrated that high signal-to-noise ratios as in our study yield in a resolution in the range of 1–2 mm [14]. Accuracy in dipole localization is affected by, for example, head movements during data acquisition, errors in wrongly specified head-models, or by errors in coregistration of anatomical images [27]. In order to minimize these systematic errors we assessed changes in the functional organization of SI by computing distance changes between cortical representations of D2 and D5. In the current experiment, both fingers were stimulated in one block. Thus, this type of errors similarly affected both dipole sources. Since we calculated the difference between dipole sources, the impact of these errors was eliminated, resulting in an improved accuracy in dipole source localization.

Similarly to the result of Petkova and Ehrsson [5], question 9 was answered positively by most of the participants. Question 9 asks if participants had perceptions that the rubber hand began to resemble their own real hand in terms of shape, skin tone, freckles or some other visual feature. In a psychometric approach Longo et al. [28] examined the qualitative responses to the rubber hand illusion by using a principal component analysis. One of the four resulting major components was called ‘affect’ and included items similar to question 9. The scores for this component were independent from synchronous and asynchronous conditions. Thus, this component can be interpreted as a measure of suggestibility.

While this study induced referred sensations or phantom sensations in healthy participants, studies with upper limb amputees similarly report referred sensations, which may even cross the body midline [17], [29]. It has been hypothesized that reorganizations in SI and in the posterior parietal cortex may cause those referred sensations [7], [8]. However, it remains an open question whether the referred touch reported in the study by Petkova and Ehrsson [5] or our study and referred sensations in upper limb amputees are based on similar neural mechanisms. Nevertheless, since the rubber hand paradigm has been used successfully to alter phantom feelings in amputees [30], future research might help us to use this knowledge in neurorehabilitation.
